# Molecular and epidemiological characterization of carbapenem-resistant hypervirulent *Klebsiella pneumoniae* in Huaian, China (2022–2024): a retrospective study

**DOI:** 10.3389/fcimb.2025.1569004

**Published:** 2025-06-04

**Authors:** Jianchun Lian, Qianhui Li, Cheng Peng, Tao Lin, Hong Du, Chaogui Tang, Xiaoyun Zhang

**Affiliations:** ^1^ Department of Clinical Laboratory, The Affiliated Huaian No.1 People’s Hospital of Nanjing Medical University, Huaian, Jiangsu, China; ^2^ Department of Clinical Laboratory, The Second Affiliated Hospital of Soochow University, Suzhou, Jiangsu, China

**Keywords:** carbapenem-resistant hypervirulent *Klebsiella pneumoniae*, multidrug resistance, molecular characterization, epidemiological characterization, risk factors, phylogenetic analysis

## Abstract

**Objectives:**

Carbapenem-resistant hypervirulent *Klebsiella pneumoniae* (CR-hvKP) poses a significant public health challenge. This study investigated the molecular epidemiology, antimicrobial resistance patterns, clinical characteristics, and risk factors of CR-hvKP infection in Huaian, China.

**Methods:**

We retrospectively studied patients infected with carbapenem-resistant *K. pneumoniae* (CRKP) between November 2022 and September 2024. Whole-genome sequencing was used to detect carbapenemase, virulence, capsular serotype-related genes, and plasmid types in 374 CRKP isolates.

**Results:**

Among them, 57.49% (215/374) strains met the criteria for CR-hvKP. The most common type was blaKPC-2-producing ST11(98.60%, 212/215), whereas K64 (56.74%, 122/215) and KL25 (39.53%, 85/215) were the main capsular serotypes. The CR-hvKP strains showed significantly higher resistance to the tested antibiotics, except for ceftazidime/avibactam and colistin. Resistance rates of CR-hvKP to the three tested antibiotics (minocycline, cotrimoxazole, and amikacin) were higher than those of CRnon-hvKP. Phylogenetic analysis based on whole-genome single-nucleotide polymorphisms divided the 251 isolates into four independent branches, with branch 2 being the most prevalent, indicating high clonality among the strains. Multivariate analysis showed diabetes [odds ratio (OR) = 3.771] and surgery (OR =2.042) to be independent variables associated with CR-hvKP infection.

**Conclusions:**

Notably, the ST11 lineage carrying blaKPC-2 has emerged as a dominant high-risk clone in Huaian. Given the wide distribution of these novel CR-hvKP isolates, global monitoring and stricter control measures should be implemented to prevent their further spread in hospital settings.

## 1 Introduction

*Klebsiella pneumoniae (K. pneumoniae)* is an opportunistic Gram-negative pathogen that can cause a range of infections, including pneumonia, urinary tract infections, bacteremia, meningitis, endophthalmitis, and pyogenic liver abscesses, posing a significant threat to public health (Jones, 2010; Martin and Bachman, 2018). Based on its virulence level (pathogenicity), it can be classified into hypervirulent *K. pneumoniae* (hvKP) and classical *K. pneumoniae* (cKP). Since the first report of hvKP in Taiwan Province in the 1980s, it has become another prevalent strain, in addition to classical cKP (Liu et al., 1986). hvKP strains can spread to distant sites, such as the lungs, liver, kidneys, spleen, fascia, eyes, and central nervous system, causing pyogenic liver abscesses and other invasive syndromes. Due to the rapid progression of infection, patients with hvKP infection usually have a poor prognosis (Fung et al., 2012; Zafar et al., 2019). In most previous studies, hvKP strains were sensitive to most antibiotics. However, owing to the spread of mobile genetic elements encoding virulence genes and carbapenemases, an increasing number of carbapenem-resistant hvKP isolates have been reported (Martin et al., 2017; Gu et al., 2018; Peng et al., 2022; Zhou et al., 2023). There are three main evolutionary patterns of carbapenem-resistant hypervirulent *K. pneumoniae*: KL1/KL2-hvKP strains acquire carbapenem-resistant plasmids and evolve into carbapenem-resistant hypervirulent *K. pneumoniae* (CR-hvKP); CRKP strains acquire virulence plasmids and transform into hv-CRKP; and cKp acquires mixed plasmids containing both carbapenem resistance genes and virulence genes (Cejas et al., 2014; Gu et al., 2018; Jin et al., 2021). Recently, reports of CR-hvKP infections have increased, with China having the highest prevalence rate (Li et al., 2014; Yao et al., 2015; Zhang et al., 2015, 2016; Wu et al., 2017; Zhan et al., 2017). which ranges from 0 to 25.8%, with a large number of infections occurring in Henan and Shandong (Zhang et al., 2020b). The results of several studies have shown that CR-hvKP has high infectivity, drug resistance, and virulence, and is easily spread in clinical environments, leading to fatal outbreaks. Therefore, these organisms have the potential to become the next “superbugs” (Jin et al., 2021), further complicating clinical practice and possibly leading to the next clinical crisis; this poses a serious threat to human health, which has attracted worldwide attention (Lee et al., 2017). Thus, public health efforts have begun to emphasize the containment of CR-hvKP transmission. This urgently requires an understanding of the geographical distribution and microbiological and molecular epidemiological characteristics of CR-hvKP infection.

In early studies, the definition of hvKP relied on a positive string test (producing a 5-millimeter-long sticky string using an inoculation loop from a bacterial colony). However, the correspondence between the string test and the clinical features of hvKP infections varied, ranging from as low as 51% to 98%. In contrast, among presumed cKp isolates, the reported positive rates of the string test were 17% and 23% (Fang et al., 2004; Lee et al., 2006; Yu et al., 2008; Lee et al., 2010; Lin et al., 2011). This indicates that the string test has poor specificity and sensitivity and cannot be used to define hvKp strains. A recent study demonstrated that the production of quantitative iron carriers ≥ 30 μg/mL, *iroB*, *iucA*, *peg-344*, *rmpA*, and *rmpA2* has been proven to have high diagnostic accuracy in identifying hvKP (Russo et al., 2018), which has been widely used in hvKP definition and research.

Although the incidence of CR-hvKP strains in China is continuously increasing, there have been relatively few epidemiological studies of CR-hvKP. Little is known about the clinical transmission and evolution of CR-hvKP, which hinders the effective intervention and control of fatal infections caused by these stubborn pathogens. This study aimed to analyze the drug resistance, clinical characteristics, molecular features, and risk factors of CR-hvKP-related infections in the Huaian region from 2022 to 2024, including genomic analysis on CR-hvKP strains to better elucidate their virulence and antimicrobial resistance mechanisms. The results of this study will help to understand the prevalence of CR-hvKP in the Huaian region, guide rational drug use, and provide a scientific basis for the prevention and control of this disease.

## 2 Materials and methods

### 2.1 Strains and identification

From November 2022 to September 2024, 374 clinical CRKP strains were collected at Huaian First People’s Hospital Affiliated to Nanjing Medical University. This hospital is a tertiary grade-A general hospital dedicated to providing medical education and has nearly 3,000 beds. All CRKP strains were continuously collected, and duplicate samples were removed based on patient information. All *K. pneumoniae* strains were identified using the MALDI-TOF MS system (bioMerieux, France), and antibiotic susceptibility testing was performed using the VITEK2 system (bioMerieux). According to the breakpoints of the Clinical and Laboratory Standards Institute (CLSI) guidelines, CRKP was defined as clinical strains that were not sensitive to carbapenems (including imipenem and meropenem). We also collected clinical data of CRKP-infected patients, including basic demographic information, length of hospital stay, underlying diseases, antibiotic exposure history, specimen type, clinical manifestations, use of invasive devices, treatment process, and outcome. We screened for virulence genes carried on plasmids to check for the presence of virulence plasmids in CRKP isolates. Plasmids containing *iucA* were defined as putative virulence plasmids because the presence of these genes is sufficient to confer a certain degree of high virulence to the host strain (Russo et al., 2018). Strains containing putative virulence plasmids were defined as CR-hvKP.

### 2.2 Antibiotic susceptibility testing

Antibiotic susceptibility testing (AST) of CRKP strains was further conducted by the broth microdilution method. Antibiotics included aztreonam, piperacillin/tazobactam, ticarcillin/clavulanic acid, cefoperazone/sulbactam, ceftazidime, imipenem, meropenem, cefepime, cotrimoxazole, minocycline, colistin, ciprofloxacin, levofloxacin, ceftazidime/avibactam, and amikacin. Data were interpreted using CLSI breakpoints (CLSI, 2021). The susceptibility to colistin was determined according to the European Committee on Antimicrobial Susceptibility Testing colistin breakpoints (EUCAST, 2021). The breakpoints for cefoperazone/sulbactam were referred to as cefoperazone in CLSI (Jones et al., 1987). The strains used for quality control were *Escherichia coli* ATCC 25922 and *K. pneumoniae* ATCC700603 (National Institute for the Control of Pharmaceutical and Biological Products, Beijing).

### 2.3 Whole genome sequencing and bioinformatics analysis

Whole-genome sequencing was performed on all 374 confirmed CRKP strains. Bacterial genomic DNA was isolated from 374 isolates using the Omega Bio-Tek Bacterial DNA Kit (Doraville, GA, USA), and concentration and purity were measured by ultraviolet spectrophotometry. Draft genome sequencing of all 374 isolates was conducted using a paired-end library with an average insert size of 350 bp on a NovaSeq 6000 sequencer (Illumina, San Diego, CA, USA), and the corrected reads were assembled *de novo* using SPAdes 3.11. Sequence types (STs), virulence-related genes, antimicrobial resistance genes, and capsular (KL) serotypes were determined using Kleborate 2.1.0.16. A Kleborate match confidence threshold of “high” or above was applied to determine whether the strain’s KL type belonged to a known serotype. Kleborate represents a novel bioinformatics tool specifically designed to assess the virulence potential of hvKP isolates through a standardized scoring system. This tool employs a 6-tiered virulence scoring metric (0-5) based on the presence or absence of three key siderophore-associated virulence determinants (Lam et al., 2021). Resistance genes were further identified using the CARD database with threshold set at ≥90% nucleotide identity and ≥80% coverage. PlasmidFinder was used to define the plasmid replicon types (http://www.genomicepidemiology.org).

### 2.4 Phylogenetic analysis

Phylogenetic analysis was performed using the complete genome sequence of *K. pneumoniae* strain HS11286 (GenBank: NC_016845.1) as the reference sequence, obtained through third-generation sequencing. The genomic sequences of target strains and outgroup strains were aligned to this reference sequence using Mummer 3.25 (Delcher et al., 2003). Core single-nucleotide polymorphism (SNP) sites were identified from the target strain group, and corresponding SNP loci from the outgroup strains were extracted. A maximum likelihood (ML) phylogenetic tree was constructed based on the core SNP sites using MEGA X 10.1.8 with a bootstrap iteration of 1000 (Kumar et al., 2018). Subsequently, the phylogenetic tree was modified using the iTOL website (Letunic and Bork, 2024).

### 2.5 *Galleria mellonella* (*G. mellonella*) killing assay

*G. mellonella* larvae were used to assess bacterial virulence (Tsai et al., 2016). Eight different sequence type (ST) clone strains of KL serotypes (HD13953, ST23, KL1; HD14933, ST11, KL156; HD12167, ST65, KL2; HD14926, ST380, KL2; HD13914, ST11, KL21; HD12319, ST11, KL25; HD12287, ST11, KL47; HD14721, ST11, KL64) were selected. Saline was used as the negative control. The hypervirulent *K. pneumoniae* reference strain NTUH-K2044 (KL1 capsular type), originally isolated from a pyogenic liver abscess and extensively characterized in virulence studies, served as the positive control (Fang et al., 2004; Russo et al., 2018). The bacterial suspension was washed twice with saline, and the *G. mellonella* larvae were randomly divided into groups of 20 larvae. Each larva was injected with a bacterial solution (10^5^ CFU), whereas the control group was injected with saline solution. Larvae that were unresponsive to stimuli were considered dead, and mortality was recorded every 12 h for 84 h. The experiment was repeated three times with a total of 60 larvae per group.

### 2.6 Statistical analysis

All data were analyzed using IBM SPSS Statistics (version 20.0; SPSS Inc., Chicago, IL, USA). Measurement data are expressed as mean ± standard deviation (SD), and count data are expressed as percentages. T-tests were performed for continuous variables. Chi-squared or Fisher’s exact tests were used for categorical variables. A logistic regression analysis was performed to further determine the risk factors for CR-hvKP infection. All statistically significant variables in the univariate analysis were included in the multivariate analysis. A *P* value < 0.05 was considered statistically significant.

## 3 Results

### 3.1 Clinical and molecular characteristics of CR-hvKP strains

Between November 2022 and September 2024, 374 non-repetitive CRKP isolates were collected from infected patients who were insensitive to at least one carbapenem, including imipenem and meropenem. The majority of the isolates were from respiratory tract specimens, such as sputum (58.56%, 219/374), followed by bronchoalveolar lavage fluid (13.10%, 49/374), urine (9.89%, 37/374), blood (6.68%, 25/374), and wound swabs (5.08%, 19/374), etc. The specimens were obtained from various hospital departments, including the intensive care unit (ICU), neurosurgery, orthopedic burn surgery, emergency care unit, neurology department, respiratory care unit, and rehabilitation medicine. Among them, Intensive Care Unit (43.32%, 162/374) and Neurosurgery (16.84%, 63/374) had the highest proportions (**Supplementary Table S1**). The CRKP strains were classified as CR-hvKP or CR-non-hvKP based on whether they carried the *iucA* virulence gene. Virulence gene detection results showed that 57.49% (215/374) of the strains were positive for *iucA*, indicating the presence of virulence plasmids belonging to the CR-hvKP group, whereas the remaining 42.51% (159/374) strains were negative for virulence genes and belonged to the CR-non-hvKP group (**Supplementary Table S1**).

Whole-genome sequencing analysis revealed that most CR-hvKP isolates belonged to the ST11 clone (99.07%, 213/215). ST15 (77.36%, 123/159) was dominant in the CR-non-hvKP strains. K64 and K25 are two serotypes closely related to CR-hvKP, with K64 (56.74%, 122/215) being the most prevalent, followed by K25 (39.53%, 85/215), and the other KL types (3.72%, 8/215). In the CR-non-hvKP strains, the capsular serotype KL19 was dominant (77.99%, 124/159) (**Table 1**). Kleborate virulence scoring revealed that among CR-hvKP strains, 212 (98.60%) achieved the score of 4, while two strains (0.93%) scored 5, and one strain (0.47%) scored 3. In contrast, the majority of CR-non-hvKP strains (151/159, 94.97%) scored 1, with only eight strains (5.03%) scoring 0.

**Table 1:** Microbiological and clinical characteristics of CR-hvKP strains.

Characteristic	CR-hvKP (n =215)	CR-non-hvKP (n =159)	*P*-value
Microbiological characteristics			
MLST			**<0.0001^b^**
ST11	212 (98.60)	14 (8.81)	
ST15	0 (0)	124 (77.99)	
Others types	3 (1.40)	21 (13.21)	
Capsular serotype			**<0.0001^b^**
KL64	120 (55.81)	14 (8.81)	
KL25	85 (39.53)	1 (0.63)	
KL19	0 (0)	124 (77.99)	
Others types	10 (4.65)	20 (12.58)	
Virulence score			
5	2 (0.93)	0 (0)	**<0.0001^b^**
4	212 (98.60)	0 (0)	
3	1 (0.47)	0 (0)	
1	0 (0)	151 (94.97)	
0	0 (0)	8 (5.03)	
Bla_Carb			**<0.0001^b^**
bla_KPC-2_	215 (100)	142 (89.31)	
bla_NDM-1_	0 (0)	6 (3.77)	
bla_OXA-48_	0 (0)	8 (5.03)	
bla_NDM-5_	0 (0)	3 (1.89)	
Number of resistance genes			**<0.0001^b^**
1-4	26 (12.09)	15 (9.43)	
5-10	126 (58.60)	124 (77.99)	
>10	63 (29.30)	20 (12.58)	
Clinical characteristics			
Basic demographics			
Age^a^, years	62.66 ± 16.00	65.71 ± 17.91	0.0874
Male	151(70.23)	107(67.30)	0.5438
Underlying diseases			
Pulmonary disease	176 (81.86)	133 (83.65)	0.6520
Diabetes mellitus	95 (44.19)	29 (18.24)	**<0.0001^b^**
Hypertension	122 (56.74)	88 (55.35)	0.7876
Cerebrovascular disease	141 (65.58)	101 (63.52)	0.6803
Cardiovascular disease	59 (27.44)	55 (34.59)	0.1376
Malignancy	22 (10.23)	17 (10.69)	0.8858
Digestive system disease	68 (31.63)	58 (36.48)	0.3266
Kidney disease	89 (41.40)	57 (35.85)	0.2771
Liver abscess	12 (5.58)	7 (4.4)	0.6078
Invasive procedures and devices			
Surgery within 1 month	153 (71.16)	91 (57.23)	**0.0052^b^**
Mechanical ventilation	118 (54.88)	85 (53.46)	0.7845
Central venous catheter	19 (8.84)	17 (10.69)	0.5477
Urinary catheter	43 (20)	34 (21.38)	0.7435
Fiberoptic Bronchoscopy	37 (17.21)	28 (17.61)	0.9195
Gastric tube	35 (16.28)	36 (22.64)	0.1209
Therapy			
Antibiotic combination therapy	110 (51.16)	71 (44.65)	0.2131
History of hormone use	173 (80.47)	133 (83.65)	0.4302
History of immunosuppressant use	12 (5.58)	12 (7.55)	0.4431
Outcomes			0.2140
Stable, discharged	161 (74.88)	131 (82.39)	
Worse, discharged	45 (20.93)	24 (15.09)	
In-hospital mortality	9 (4.19)	4 (2.52)	
Length of stay ^a^, days	33 ± 27.72	36.27 ± 29.81	0.2802

Continuous variables (Age and Length of stay) were compared using Student’s t-test; other variables were analyzed by Chi-square test (or Fisher’s exact test when expected cell counts were <5). All p-values were two-tailed, and significance was set at *P*< 0.05. a Age and length of stay are presented as mean and standard deviation (SD). ^b^ Bold font means *P* < 0.05. CR-hvKP, carbapenem-resistant hypervirulent *K. pneumoniae*; MLST, multilocus sequence type; ST, Sequence Type.

Whole-genome sequencing revealed that all CR-hvKP isolates carried the bla_KPC-2_ carbapenemase gene (100.00%, 215/215). In CR-non-hvKP isolates, bla_KPC-2_, bla_NDM-1_, bla_NDM-5_, and bla_OXA-48_ carbapenemases were detected, with bla_KPC-2_ being the predominant carbapenemase (89.31%, 142/159), followed by bla_OXA-48_ (5.03%, 8/159), bla_NDM-1_ (3.77%, 6/159), and bla_NDM-5_ (1.89%, 3/159). Both CR-hvKP and CR-non-hvKP carried multiple resistance genes (**Table 1**).

### 3.2 Susceptibility results and carbapenemase resistance genes

All CR-hvKP and CR-non-hvKP strains were resistant to imipenem, and only two CR-non-hvKP strains were sensitive to meropenem. All the strains showed high resistance to cephalosporins, monobactams, aminoglycosides, tetracyclines, β-lactam-β-lactamase inhibitor combinations, and fluoroquinolones. However, the resistance rates to ceftazidime/avibactam and colistin were relatively low, 1.40% and 12.09%, respectively. The rates of resistance to sulfamethoxazole/trimethoprim, minocycline, and amikacin in the CR-hvKP group were significantly higher than those in the CR-non-hvKP infection group (*P* < 0.05). The resistance rate to ceftazidime/avibactam in the CR-hvKP infection group was significantly lower than that in the CR-non-hvKP infection group (*P* < 0.05) (**Table 2**).

**Table 2:** Susceptibility of CR-hvKP and CR-non-hvKP strains against different antimicrobial agents.

Antibiotic agents	CR-hvKP (n =215)		CR-non-hvKP (n = 159)		*P*-value
	Number	Resistance (%)	Number	Resistance (%)	
Aztreonam	214	99.53	155	97.48	0.1678
Piperacillin/Tazobactam	214	99.53	157	98.74	0.5771
Ticacillin/clavulanic acid	214	99.53	157	98.74	0.5771
Cefoperazone/Sulbactama	210	99.67	151	94.97	0.2526
Ceftazidime	206	95.81	157	98.74	0.1263
Imipenem	215	100	157	98.74	0.1801
Meropenem	215	100	159	100	/
Cefepime	214	99.53	157	98.74	0.5771
Sulfamethoxazole	106	49.3	48	30.19	**0.0002^a^**
Minocycline	192	89.3	119	74.84	**0.0002^a^**
Colistin	26	12.09	25	15.72	0.6544
Ciprofloxacin	213	99.07	156	98.11	0.4259
Levofloxacin	212	98.6	156	98.11	0.7022
Amikacin	150	69.77	34	21.38	**<0.0001^a^**
Ceftazidime-avibactam	3	1.4	9	5.66	**0.0338^a^**

Categorical variables were analyzed by the Chi-square test (or Fisher’s exact test when expected cell counts were <5). All *P*-values were two-tailed, and significance was set at *P* < 0.05. a Bold font means *P* < 0.05.

### 3.3 Plasmids

**Figure 1:**
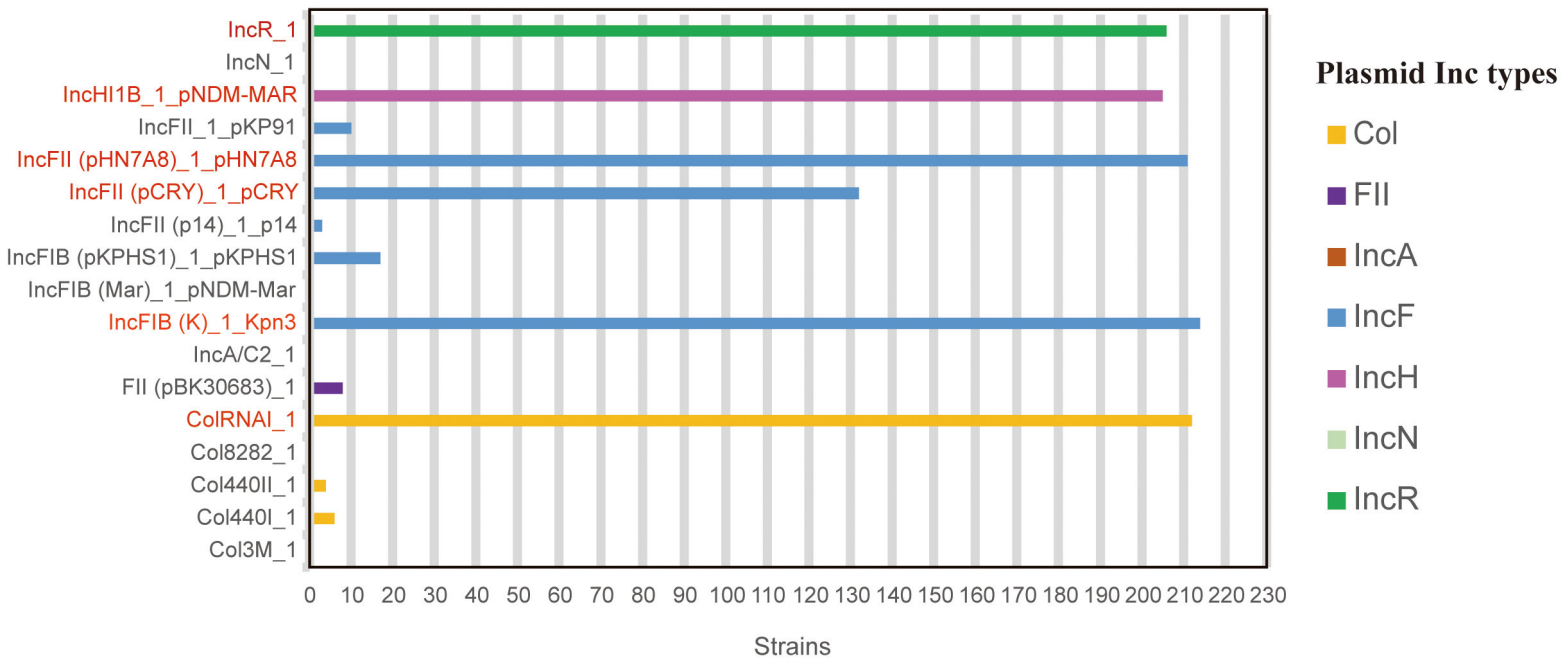
Distribution of plasmid types among 215 CR-hvKP strains. Data derived from whole-genome sequencing analysis of 215 CR-hvKP isolates. Plasmid types were identified using PlasmidFinder (threshold: >95% identity, >80% coverage). The six most prevalent plasmids (detection rate >60%) are highlighted in red, others in gray.

A total of 17 plasmids (seven families) were detected in 215 strains. IncF plasmids were the most diverse (seven subtypes). Among the 17 plasmids, IncFIB(K)_1_Kpn3 (99.53%, 214/215), ColRNAI_1 (98.60%, 212/215), IncFII(pHN7A8) _1_pHN7A8 (98.14%, 211/215), IncR_1 (95.81%, 206/215), IncHI1B_1_pNDM-MAR (95.35%, 205/215), and IncFII (pCRY)_1_pCRY (61.40%, 132/215) were the most common, while the occurrence rates of other plasmids ranged from 0.47% to 7.91% (**Figure 1**).

### 3.4 Phylogenetic analysis

**Figure 2:**
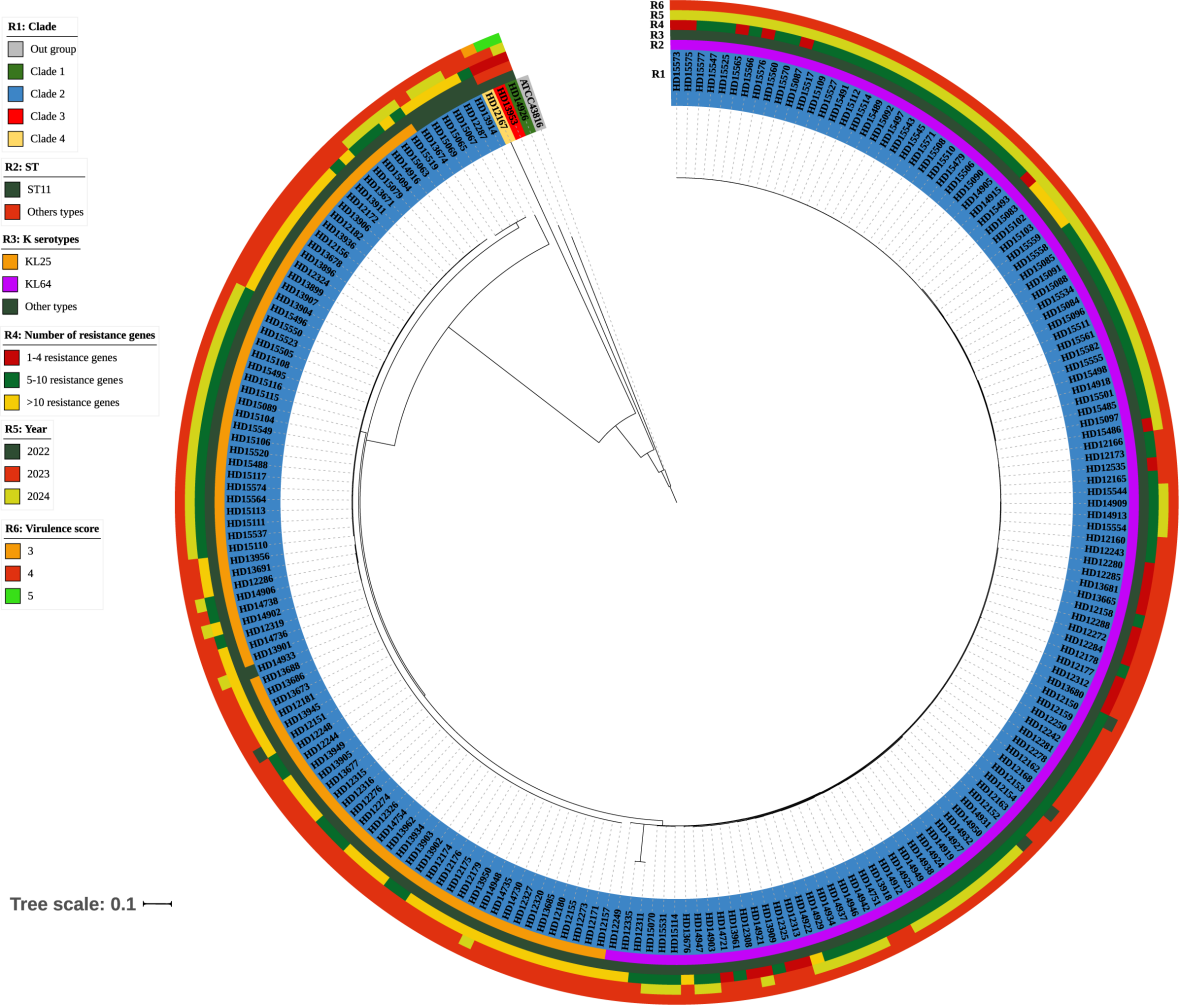
Phylogenetic analysis of 215 genomes of CR-hvKP. A maximum likelihood phylogenetic tree was constructed using the core genes of 215 *K. pneumoniae* isolates. The colors of the branches correspond to clades. Strips illustrate strain number, ST, KL types, number of resistance genes, year, and virulence score from inside to outside. The bar corresponds to the scale of sequence divergence.

Phylogenetic analysis of whole-genome single-nucleotide polymorphisms was conducted on 215 CR-hvKP isolates, identifying four major branches, designated as branches 1-4 (**Figure 2**). Branches 1, 3, and 4 were mainly composed of relatively small proportions of ST65, ST23, and ST380 clones, and each branch contained only one strain, mainly KL1 (33.33%, 1/3) and KL2(66.67%, 2/3), isolated in 2023 and 2024, respectively. The remaining 212 strains formed branch 2, which comprised ST11 clones. Branch 2 mainly comprised KL64 (56.74%, 122/215) and KL25 (39.53%, 85/215), isolated from 2022 to 2024, with most strains carrying five or more resistance genes (**Figure 2**).

### 3.5 *Galleria mellonella* (*G. mellonella*) infection

**Figure 3:**
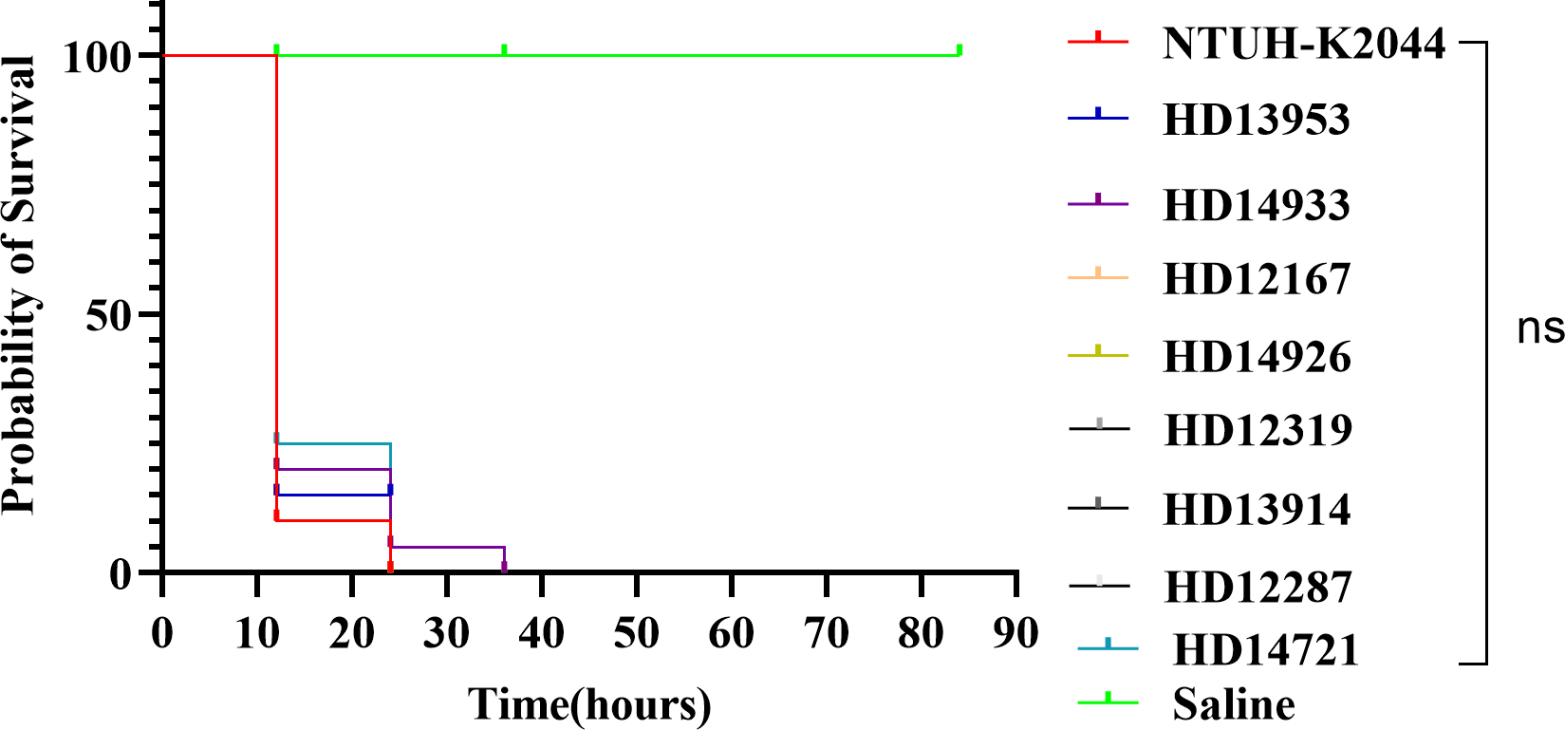
Survival rates of larvae infected with eight distinct ST/KL-type CR-hvKP strains. There was no statistical difference in the survival rates between NTUH-K2044 and the eight CR-hvKP strains. A log-rank (Mantel-Cox) test was performed for the survival curves.

The *in vivo* virulence of 8 CR-hvKP strains was initially evaluated using a *G. mellonella* killing assay. The strains were injected into *G. mellonella* larvae, and the survival rate at a specific time point was used as a virulence index. After 24 h of infection, except for HD14933 and HD12287, the survival rate of larvae treated with NTUH-K2044 or the other 6 CR-hvKP strains was 0%. The survival rate of larvae infected with HD14933 and HD12287 was 0% at 36 h. There was no statistical difference in the survival rates between NTUH-K2044 and the eight CR-hvKP strains. These results indicate that the virulence of the eight CR-hvKP strains was approximately the same as that of NTUH-K2044, with high virulence and strong pathogenicity (**Figure 3**).

### 3.6 Clinical factors of CR-hvKP infection

The demographic and clinical factors of the patients with CR-hvKP and CR-non-hvKP infections are summed up in **Table 1**. Among the 215 patients, the average age was 62.66 ± 16.00 years, and 70.23% (151/215) were male. The average hospital stay day after CR-hvKP and CR-non-hvKP infections was 33 ± 27.72 days and 36.27 ± 29.81 days, respectively. The main comorbidities of all infected patients mainly involved pulmonary diseases (81.86%, 176/215), diabetes (44.19%, 95/215), hypertension (56.74%, 122/215), cerebrovascular diseases (65.58%, 141/215), kidney diseases (41.40%, 89/215), digestive system diseases (31.63%, 68/215), and cardiovascular diseases (27.44%, 59/215). There were no significant differences between the CR-hvKP group and CR-non-hvKP group in terms of age, sex, antibiotic exposure, and length of hospital stay from CRKP isolation to outcome (in-hospital death or discharge) (*P* > 0.05). Additionally, except for diabetes (44.19% vs. 18.24%, P < 0.0001), there were no significant differences in most underlying diseases, such as pulmonary diseases, cardiovascular diseases, hypertension, cerebrovascular diseases, malignant tumors, and liver abscesses (*P* > 0.05). The rate of patients who underwent surgery within one month was higher in the CR-hvKP group than in the CR-non-hvKP group (71.16% vs. 57.23%, *P* = 0.0052), whereas other invasive procedures had no significant differences (**Table 1**).

### 3.7 Risk factors for CR-hvKP infection

Two variables from the univariate analysis (*P* < 0.05) were included in the multivariate analysis: diabetes and surgery. The results showed that diabetes (OR =3.771; *P* < 0.0001) and surgery (OR =2.042; *P* = 0.0021) were independent predictors of CR-hvKP infection in patients (**Table 3**).

**Table 3:** Univariate and multivariate logistic regression analysis of CR-hvKP infections.

Variables	Univariate analysis		Multivariable analysis	
	OR (95% CI)	*P*-value	OR (95% CI)	*P*-value
Diabetes mellitus	3.549 (2.209 to 5.833)	<0.0001^a^	3.771 (2.326 to 6.265)	<0.0001^a^
Surgery within 1 month	1.844 (1.200 to 2.844)	0.0054^a^	2.042 (1.300 to 3.230)	0.0021^a^

a Bold font means *P*< 0.05. OR, Odds Ratio.

## 4 Discussion

The occurrence of hvKP was first reported in China more than 30 years ago (Liu et al., 1986). In recent years, with the widespread use of antibiotics and the intensification of global interpersonal communication, reports on CR-hvKP have gradually increased. Because of its high virulence and resistance to multiple drugs, including carbapenems, it can spread widely in clinical settings and cause fatal outbreaks (Gu et al., 2018; Yang et al., 2021). It is one of the main causes of death in patients with hospital-acquired infections, and poses a significant threat to global public health. To understand the molecular epidemiological characteristics of CR-hvKP in Huaian, this study investigated the prevalence of CR-hvKP strains isolated from the clinical specimens of patients in Huaian and described the drug resistance, molecular characteristics, and clinical risk factors of these isolates.

Previous studies have found that hvKP infection occurs at unusual (sometimes multiple) sites and is thought to be associated with diseases, such as liver abscesses and invasive eye inflammation, often accompanied by bacteremia and metastatic spread (Prokesch et al., 2016; Li et al., 2018; Wyres et al., 2020). However, the main specimen type of CR-hvKP strains isolated in this study was sputum, and only 5.58% of patients with CR-hvKP infection had symptoms of liver abscess. It can be seen that hvKP is associated with various infections, including bacteremia, pneumonia, soft tissue infection, and so on (Russo and Marr, 2019). The proportion of CR-hvKP in ICU patients was significantly higher than in other departments. We speculate that patients in the ICU are at a higher risk of CR-hvKP infection, which may be related to changes in the patient’s immune status, significantly higher antibiotic exposure than that in other departments, and more invasive procedures during treatment.

*K. pneumoniae* can resist the effects of antimicrobial drugs through various mechanisms, including target alteration, drug inactivation, enhanced efflux pump activity, and decreased cell permeability. Carbapenemase production is the most common mechanism of carbapenem resistance in the CRKP strains (Zhang et al., 2017). According to data from the Chinese Antimicrobial Resistance Surveillance System (http://www.carss.cn), the prevalence of CRKP in central provinces of China in recent years ranged from 0.60% to 26.20%, with an average of 10.80%. The main carbapenem-resistant genotype of CRKP is bla_KPC_, accounting for approximately 65.00% of cases (Han et al., 2020). Similarly, all 215 CR-hvKP strains in this study carried bla_KPC-2_, whereas only some CR-non-hvKP strains carried bla_OXA-48_, bla_NDM-1_, or bla_NDM-5_. The CR-hvKP strains showed high resistance to drugs such as carbapenems, aminoglycosides, fluoroquinolones, tetracyclines, and cephalosporins. Caution should be exercised when using antibiotics to treat CR-hvKP infections. Fortunately, CR-hvKP isolates showed low resistance to colistin and ceftazidime/avibactam, 12.09% and 1.40%, respectively. CZA is an effective option for treating CR-hvKP infections that produce bla_KPC_ or bla_OXA-48_ (Mataraci Kara et al., 2020; Mehmood et al., 2020). Metallo-β-lactamase (MBL) production is a common cause of ceftazidime/avibactam resistance in China, and other possible causes may be OmpK35/36 deficiency, bla_KPC-2_ point mutations, or higher bla_KPC-2_ copy number and gene expression (Cui et al., 2020; Zhang et al., 2020a). The comparative analysis using the CARD database revealed that all 357 CRKP strains in this study carried the wild-type blaKPC-2 without any point mutations. Resistance to ceftazidime-avibactam (CZA) in 9 CR-non-hvKP strains was mediated by MBL production (NDM-1 and NDM-5). For the three CR-hvKP strains, the observed CZA resistance may be attributed to elevated blaKPC-2 copy numbers and upregulated expression levels, which require further experimental confirmation. Strikingly, we found that CR-hvKP strains had higher resistance rates to three antimicrobial drugs, minocycline, amikacin, and cotrimoxazole, compared with CR-non-hvKP strains, which may be a key consideration in the clinical management of CR-hvKP infection.

There were 33 ST and 15 capsular serotypes among the 215 CR-hvKP isolates in this study. Notably, among these CR-hvKP strains, ST11 type strains dominated (99.07%, 213/215), indicating that ST11 CRKP strains were most likely the leading cause of the acquisition and spread of virulence plasmids. In addition to the virulence-related plasmids, the capsule is the main factor responsible for the virulence of CR-hvKP. K64 (wzi-64) in *K. pneumoniae* has been identified as a common CRKP strain in China and other countries (Koh et al., 2013; Zhou et al., 2020). In this study, K64 was the major capsular serotype (56.74%, 122/215), followed by KL25 (39.53%, 85/215). We speculate that the ST11-K64 and ST11-K25 CR-hvKP subclones producing bla_KPC-2_ spread throughout the Huaian area. More alarmingly, studies have found that a fatal outbreak caused by ST11 CR-hvKP occurred in the ICU, resulting in poor prognosis for all infected patients (Jiang et al., 2015). Notably, the virulence scores of CR-hvKP strains were high in our study. All eight CR-hvKP strains of different ST/KL types exhibited high virulence levels, showing no significant difference from the high-virulent reference strain NTUH-K2044. The emergence of bla_KPC_-producing ST11 CR-hvKP may pose a serious challenge to managing patients infected with these strains.

Plasmids are considered the primary source of *K. pneumoniae* gene variation and have been used as molecular markers in epidemiological investigations (Carattoli et al., 2014). *K. pneumoniae* has multiple replicon types, which are classified into discrete incompatibility (inc) groups or families based on the inability of closely related plasmids to coexist stably within the same bacterial strain (Yao et al., 2020). IncF family plasmids, widely present in Enterobacteriaceae, are characterized by their association with multidrug resistance (Johnson and Nolan, 2009), and significantly contribute to *K. pneumoniae* drug resistance. The differences in plasmid structure and content among strains isolated from the same hospital suggest the possible emergence of additional plasmid types and arrangements that require close monitoring. The characteristics of the plasmids and the drug resistance and virulence genes they carry require further study. The phylogenetic analysis indicated that the 215 CR-hvKP strains could be classified into four major branches: 1, 2, 3, and 4, with branch 2 being dominant. Strains with the same ST are more closely related and often carry the same virulence and antibiotic resistance genes. This indicates an urgent need for stricter monitoring and infection control measures to prevent the spread of clonal strains in hospital environments.

Patients with CR-hvKP infection have severe symptoms and poor prognosis, especially in elderly, pediatric, and immunocompromised patients. Therefore, a better understanding of the risk factors for CR-hvKP infection is urgently required. In this study, we evaluated risk factors associated with CR-hvKP isolates. Univariate and multivariate analyses revealed that diabetes was an independent predictor of CR-hvKP infection. It is consistent with a previous retrospective study, which showed that hvKP infections mainly occur in susceptible individuals with an underlying condition of diabetes (Fazili et al., 2016). Studies found that the bactericidal ability of diabetic neutrophil extracellular traps (NETs) to hvKP was impaired, which partly illustrated why diabetics were vulnerable to hvKP infections (Jin et al., 2020). Additionally, surgery within the previous month was an independent predictor of CR-hvKP infection, which may be related to the fact that surgery can lower patient immunity and increase the risk of infection or colonization.

This study has some limitations. First, this was a single-center retrospective study rather than a multicenter longitudinal molecular epidemiological study of CR-hvKP, and the number of patients was relatively small. Second, the definition of high-virulence cases relied on laboratory-based analyses and *G. mellonella* virulence assays. A positive result for virulence genes in some strains does not necessarily mean “definite” high virulence. Combining clinical features with genotypic and phenotypic characteristics can better define CR-hvKP strains. More prospective and multicenter studies are needed to investigate the clinical characteristics of CR-hvKP and conduct whole-genome sequencing analysis to clarify the clustering, transmission mechanisms, and clinical risk factors of CR-hvKP.

## 5 Conclusion

This study involved a molecular epidemiological investigation of 374 non-repetitive CR-hvKP and CR-non-hvKP isolates from the Huaian region. The CR-hvKP strains were more prevalent in the hospital environment than the CR-non-hvKP strains. The most common carbapenemase was blaKPC-2. Diabetes and surgery within the previous month were independent predictors of CR-hvKP infection. Notably, the ST11 lineage carrying blaKPC-2 has emerged as a dominant high-risk clone in this region, which requires enhanced infection control measures to curb its transmission.

## Data Availability

The datasets presented in this study can be found in online repositories. The names of the repository/repositories and accession number(s) can be found in the article/**Supplementary Material**.
