# Supplementary material for: Molecular and epidemiological characterization of carbapenem-resistant hypervirulent Klebsiella pneumoniae in Huaian, China (2022–2024): a retrospective study
Source: Front Cell Infect Microbiol. 2025 Jun 4;15:1569004. doi: 10.3389/fcimb.2025.1569004 (PMC12174136; doi:10.3389/fcimb.2025.1569004)
Supplement: Supplementary file 1 [file Table1.docx]

Table S1 Specimen types and departments of CR-hvKP and CR-non- hvKP strains.

|  | **CR-hvKP (n =215)** | | **CR-non-hvKP**  **(n =159)** | | **Total (n=374)** | |
| --- | --- | --- | --- | --- | --- | --- |
|  | **Number** | **Percentage (%)** | **Number** | **Percentage (%)** | **Number** | **Percentage (%)** |
| Specimen types |  |  |  |  |  |  |
| Sputum | 121 | 56.28 | 98 | 61.63 | 219 | 58.56 |
| bronchoalveolar lavage fluid | 30 | 13.95 | 19 | 11.95 | 49 | 13.10 |
| Urine | 22 | 10.23 | 15 | 9.43 | 37 | 9.89 |
| Blood | 18 | 8.37 | 7 | 4.40 | 25 | 6.68 |
| Wound exudate | 9 | 4.19 | 10 | 6.29 | 19 | 5.08 |
| Cerebrospinal fluid | 4 | 1.86 | 2 | 1.26 | 6 | 1.6 |
| Drainage fluid | 4 | 1.86 | 4 | 2.52 | 8 | 2.14 |
| Purulent exudate | 2 | 0.93 | 0 | 0.00 | 2 | 0.53 |
| Throat swab | 2 | 0.93 | 0 | 0.00 | 2 | 0.53 |
| Pleural fluid | 1 | 0.47 | 0 | 0.00 | 1 | 0.27 |
| Ascitic fluid | 1 | 0.47 | 1 | 0.63 | 2 | 0.53 |
| Catheter tip | 1 | 0.47 | 1 | 0.63 | 2 | 0.53 |
| Bile | 0 | 0 | 2 | 1.26 | 2 | 0.53 |
| Departments | | | | | | |
| Intensive Care Unit (ICU) | 90 | 41.86 | 72 | 45.28 | 162 | 43.32 |
| Neurosurgery | 44 | 20.47 | 19 | 11.95 | 63 | 16.84 |
| Burn Department | 10 | 4.65 | 4 | 2.52 | 14 | 3.74 |
| Emergency Room (ER) | 10 | 4.65 | 7 | 4.40 | 17 | 4.55 |
| Neurology | 8 | 3.72 | 3 | 1.89 | 11 | 2.94 |
| Respiratory Care Unit (RCU) | 8 | 3.72 | 9 | 5.66 | 17 | 4.55 |
| Rehabilitation Department | 8 | 3.72 | 9 | 5.66 | 17 | 4.55 |
| Emergency Department (ED) | 7 | 3.26 | 2 | 1.26 | 9 | 2.41 |
| Pulmonology | 5 | 2.33 | 0 | 0.00 | 5 | 1.34 |
| Thoracic Surgery | 4 | 1.86 | 3 | 1.89 | 7 | 1.87 |
| General Surgery | 4 | 1.86 | 7 | 4.40 | 11 | 2.94 |
| Cardiac Surgery | 3 | 1.4 | 0 | 0.00 | 3 | 0.8 |
| Geriatrics | 3 | 1.4 | 12 | 7.55 | 15 | 4.01 |
| Oncology | 2 | 0.93 | 0 | 0.00 | 2 | 0.53 |
| Dental Department | 2 | 0.93 | 0 | 0.00 | 2 | 0.53 |
| Urology | 2 | 0.93 | 2 | 1.26 | 4 | 1.07 |
| Endocrinology | 1 | 0.47 | 0 | 0.00 | 1 | 0.27 |
| Nephrology | 1 | 0.47 | 0 | 0.00 | 1 | 0.27 |
| Orthopedics | 1 | 0.47 | 2 | 1.26 | 3 | 0.8 |
| Pediatrics | 1 | 0.47 | 2 | 1.26 | 3 | 0.8 |
| Rheumatology & Immunology | 1 | 0.47 | 0 | 0.00 | 1 | 0.27 |
| Gastrointestinal Surgery | 0 | 0 | 1 | 0.63 | 1 | 0.27 |
| Hematology | 0 | 0 | 3 | 1.89 | 3 | 0.8 |
| Vascular & Interventional Radiology | 0 | 0 | 1 | 0.63 | 1 | 0.27 |
| General Practice | 0 | 0 | 1 | 0.63 | 1 | 0.27 |
