# Supplementary material for: Molecular and epidemiological characterization of carbapenem-resistant hypervirulent Klebsiella pneumoniae in Huaian, China (2022–2024): a retrospective study
Source: Front Cell Infect Microbiol. 2025 Jun 4;15:1569004. doi: 10.3389/fcimb.2025.1569004 (PMC12174136; doi:10.3389/fcimb.2025.1569004)
Supplement: Supplementary file 2 [file Table2.docx]

**TableS2 Strain numbers, strain types and GenBank accession number in this study.**

| **Strain number** | **Strain type** | **GenBank accession number** |
| --- | --- | --- |
| HD12150 | CR-hvKP | BioProject PRJNA1256526 |
| HD12151 | CR-hvKP | BioProject PRJNA1256526 |
| HD12152 | CR-hvKP | BioProject PRJNA1256526 |
| HD12153 | CR-hvKP | BioProject PRJNA1256526 |
| HD12154 | CR-hvKP | BioProject PRJNA1256526 |
| HD12155 | CR-hvKP | BioProject PRJNA1256526 |
| HD12156 | CR-hvKP | BioProject PRJNA1256526 |
| HD12157 | CR-hvKP | BioProject PRJNA1256526 |
| HD12158 | CR-hvKP | BioProject PRJNA1256526 |
| HD12159 | CR-hvKP | BioProject PRJNA1256526 |
| HD12160 | CR-hvKP | BioProject PRJNA1256526 |
| HD12162 | CR-hvKP | BioProject PRJNA1256526 |
| HD12163 | CR-hvKP | BioProject PRJNA1256526 |
| HD12165 | CR-hvKP | BioProject PRJNA1256526 |
| HD12166 | CR-hvKP | BioProject PRJNA1256526 |
| HD12167 | CR-hvKP | BioProject PRJNA1256526 |
| HD12168 | CR-hvKP | BioProject PRJNA1256526 |
| HD12171 | CR-hvKP | BioProject PRJNA1256526 |
| HD12172 | CR-hvKP | BioProject PRJNA1256526 |
| HD12173 | CR-hvKP | BioProject PRJNA1256526 |
| HD12174 | CR-hvKP | BioProject PRJNA1256526 |
| HD12175 | CR-hvKP | BioProject PRJNA1256526 |
| HD12176 | CR-hvKP | BioProject PRJNA1256526 |
| HD12177 | CR-hvKP | BioProject PRJNA1256526 |
| HD12178 | CR-hvKP | BioProject PRJNA1256526 |
| HD12179 | CR-hvKP | BioProject PRJNA1256526 |
| HD12180 | CR-hvKP | BioProject PRJNA1256526 |
| HD12181 | CR-hvKP | BioProject PRJNA1256526 |
| HD12182 | CR-hvKP | BioProject PRJNA1256526 |
| HD12242 | CR-hvKP | BioProject PRJNA1256526 |
| HD12243 | CR-hvKP | BioProject PRJNA1256526 |
| HD12244 | CR-hvKP | BioProject PRJNA1256526 |
| HD12248 | CR-hvKP | BioProject PRJNA1256526 |
| HD12249 | CR-hvKP | BioProject PRJNA1256526 |
| HD12250 | CR-hvKP | BioProject PRJNA1256526 |
| HD12272 | CR-hvKP | BioProject PRJNA1256526 |
| HD12273 | CR-hvKP | BioProject PRJNA1256526 |
| HD12274 | CR-hvKP | BioProject PRJNA1256526 |
| HD12276 | CR-hvKP | BioProject PRJNA1256526 |
| HD12278 | CR-hvKP | BioProject PRJNA1256526 |
| HD12280 | CR-hvKP | BioProject PRJNA1256526 |
| HD12281 | CR-hvKP | BioProject PRJNA1256526 |
| HD12284 | CR-hvKP | BioProject PRJNA1256526 |
| HD12285 | CR-hvKP | BioProject PRJNA1256526 |
| HD12286 | CR-hvKP | BioProject PRJNA1256526 |
| HD12287 | CR-hvKP | BioProject PRJNA1256526 |
| HD12288 | CR-hvKP | BioProject PRJNA1256526 |
| HD12308 | CR-hvKP | BioProject PRJNA1256526 |
| HD12311 | CR-hvKP | BioProject PRJNA1256526 |
| HD12312 | CR-hvKP | BioProject PRJNA1256526 |
| HD12313 | CR-hvKP | BioProject PRJNA1256526 |
| HD12315 | CR-hvKP | BioProject PRJNA1256526 |
| HD12316 | CR-hvKP | BioProject PRJNA1256526 |
| HD12319 | CR-hvKP | BioProject PRJNA1256526 |
| HD12320 | CR-hvKP | BioProject PRJNA1256526 |
| HD12324 | CR-hvKP | BioProject PRJNA1256526 |
| HD12325 | CR-hvKP | BioProject PRJNA1256526 |
| HD12326 | CR-hvKP | BioProject PRJNA1256526 |
| HD12327 | CR-hvKP | BioProject PRJNA1256526 |
| HD12335 | CR-hvKP | BioProject PRJNA1256526 |
| HD12535 | CR-hvKP | BioProject PRJNA1256526 |
| HD13665 | CR-hvKP | BioProject PRJNA1256526 |
| HD13671 | CR-hvKP | BioProject PRJNA1256526 |
| HD13673 | CR-hvKP | BioProject PRJNA1256526 |
| HD13674 | CR-hvKP | BioProject PRJNA1256526 |
| HD13676 | CR-hvKP | BioProject PRJNA1256526 |
| HD13677 | CR-hvKP | BioProject PRJNA1256526 |
| HD13678 | CR-hvKP | BioProject PRJNA1256526 |
| HD13680 | CR-hvKP | BioProject PRJNA1256526 |
| HD13681 | CR-hvKP | BioProject PRJNA1256526 |
| HD13685 | CR-hvKP | BioProject PRJNA1256526 |
| HD13686 | CR-hvKP | BioProject PRJNA1256526 |
| HD13688 | CR-hvKP | BioProject PRJNA1256526 |
| HD13691 | CR-hvKP | BioProject PRJNA1256526 |
| HD13896 | CR-hvKP | BioProject PRJNA1256526 |
| HD13899 | CR-hvKP | BioProject PRJNA1256526 |
| HD13901 | CR-hvKP | BioProject PRJNA1256526 |
| HD13902 | CR-hvKP | BioProject PRJNA1256526 |
| HD13903 | CR-hvKP | BioProject PRJNA1256526 |
| HD13904 | CR-hvKP | BioProject PRJNA1256526 |
| HD13905 | CR-hvKP | BioProject PRJNA1256526 |
| HD13906 | CR-hvKP | BioProject PRJNA1256526 |
| HD13907 | CR-hvKP | BioProject PRJNA1256526 |
| HD13909 | CR-hvKP | BioProject PRJNA1256526 |
| HD13911 | CR-hvKP | BioProject PRJNA1256526 |
| HD13914 | CR-hvKP | BioProject PRJNA1256526 |
| HD13918 | CR-hvKP | BioProject PRJNA1256526 |
| HD13934 | CR-hvKP | BioProject PRJNA1256526 |
| HD13936 | CR-hvKP | BioProject PRJNA1256526 |
| HD13945 | CR-hvKP | BioProject PRJNA1256526 |
| HD13949 | CR-hvKP | BioProject PRJNA1256526 |
| HD13950 | CR-hvKP | BioProject PRJNA1256526 |
| HD13953 | CR-hvKP | BioProject PRJNA1256526 |
| HD13956 | CR-hvKP | BioProject PRJNA1256526 |
| HD13961 | CR-hvKP | BioProject PRJNA1256526 |
| HD13962 | CR-hvKP | BioProject PRJNA1256526 |
| HD14721 | CR-hvKP | BioProject PRJNA1256526 |
| HD14730 | CR-hvKP | BioProject PRJNA1256526 |
| HD14735 | CR-hvKP | BioProject PRJNA1256526 |
| HD14736 | CR-hvKP | BioProject PRJNA1256526 |
| HD14738 | CR-hvKP | BioProject PRJNA1256526 |
| HD14751 | CR-hvKP | BioProject PRJNA1256526 |
| HD14754 | CR-hvKP | BioProject PRJNA1256526 |
| HD14902 | CR-hvKP | BioProject PRJNA1256526 |
| HD14903 | CR-hvKP | BioProject PRJNA1256526 |
| HD14905 | CR-hvKP | BioProject PRJNA1256526 |
| HD14906 | CR-hvKP | BioProject PRJNA1256526 |
| HD14909 | CR-hvKP | BioProject PRJNA1256526 |
| HD14912 | CR-hvKP | BioProject PRJNA1256526 |
| HD14913 | CR-hvKP | BioProject PRJNA1256526 |
| HD14915 | CR-hvKP | BioProject PRJNA1256526 |
| HD14916 | CR-hvKP | BioProject PRJNA1256526 |
| HD14918 | CR-hvKP | BioProject PRJNA1256526 |
| HD14919 | CR-hvKP | BioProject PRJNA1256526 |
| HD14921 | CR-hvKP | BioProject PRJNA1256526 |
| HD14922 | CR-hvKP | BioProject PRJNA1256526 |
| HD14924 | CR-hvKP | BioProject PRJNA1256526 |
| HD14925 | CR-hvKP | BioProject PRJNA1256526 |
| HD14926 | CR-hvKP | BioProject PRJNA1256526 |
| HD14927 | CR-hvKP | BioProject PRJNA1256526 |
| HD14929 | CR-hvKP | BioProject PRJNA1256526 |
| HD14931 | CR-hvKP | BioProject PRJNA1256526 |
| HD14932 | CR-hvKP | BioProject PRJNA1256526 |
| HD14933 | CR-hvKP | BioProject PRJNA1256526 |
| HD14934 | CR-hvKP | BioProject PRJNA1256526 |
| HD14937 | CR-hvKP | BioProject PRJNA1256526 |
| HD14938 | CR-hvKP | BioProject PRJNA1256526 |
| HD14942 | CR-hvKP | BioProject PRJNA1256526 |
| HD14946 | CR-hvKP | BioProject PRJNA1256526 |
| HD14947 | CR-hvKP | BioProject PRJNA1256526 |
| HD14948 | CR-hvKP | BioProject PRJNA1256526 |
| HD14949 | CR-hvKP | BioProject PRJNA1256526 |
| HD14950 | CR-hvKP | BioProject PRJNA1256526 |
| HD15063 | CR-hvKP | BioProject PRJNA1256526 |
| HD15065 | CR-hvKP | BioProject PRJNA1256526 |
| HD15067 | CR-hvKP | BioProject PRJNA1256526 |
| HD15069 | CR-hvKP | BioProject PRJNA1256526 |
| HD15070 | CR-hvKP | BioProject PRJNA1256526 |
| HD15079 | CR-hvKP | BioProject PRJNA1256526 |
| HD15083 | CR-hvKP | BioProject PRJNA1256526 |
| HD15084 | CR-hvKP | BioProject PRJNA1256526 |
| HD15085 | CR-hvKP | BioProject PRJNA1256526 |
| HD15087 | CR-hvKP | BioProject PRJNA1256526 |
| HD15088 | CR-hvKP | BioProject PRJNA1256526 |
| HD15089 | CR-hvKP | BioProject PRJNA1256526 |
| HD15090 | CR-hvKP | BioProject PRJNA1256526 |
| HD15091 | CR-hvKP | BioProject PRJNA1256526 |
| HD15092 | CR-hvKP | BioProject PRJNA1256526 |
| HD15094 | CR-hvKP | BioProject PRJNA1256526 |
| HD15096 | CR-hvKP | BioProject PRJNA1256526 |
| HD15097 | CR-hvKP | BioProject PRJNA1256526 |
| HD15102 | CR-hvKP | BioProject PRJNA1256526 |
| HD15103 | CR-hvKP | BioProject PRJNA1256526 |
| HD15104 | CR-hvKP | BioProject PRJNA1256526 |
| HD15106 | CR-hvKP | BioProject PRJNA1256526 |
| HD15108 | CR-hvKP | BioProject PRJNA1256526 |
| HD15109 | CR-hvKP | BioProject PRJNA1256526 |
| HD15110 | CR-hvKP | BioProject PRJNA1256526 |
| HD15111 | CR-hvKP | BioProject PRJNA1256526 |
| HD15112 | CR-hvKP | BioProject PRJNA1256526 |
| HD15113 | CR-hvKP | BioProject PRJNA1256526 |
| HD15114 | CR-hvKP | BioProject PRJNA1256526 |
| HD15115 | CR-hvKP | BioProject PRJNA1256526 |
| HD15116 | CR-hvKP | BioProject PRJNA1256526 |
| HD15117 | CR-hvKP | BioProject PRJNA1256526 |
| HD15479 | CR-hvKP | BioProject PRJNA1256526 |
| HD15485 | CR-hvKP | BioProject PRJNA1256526 |
| HD15486 | CR-hvKP | BioProject PRJNA1256526 |
| HD15488 | CR-hvKP | BioProject PRJNA1256526 |
| HD15489 | CR-hvKP | BioProject PRJNA1256526 |
| HD15491 | CR-hvKP | BioProject PRJNA1256526 |
| HD15493 | CR-hvKP | BioProject PRJNA1256526 |
| HD15495 | CR-hvKP | BioProject PRJNA1256526 |
| HD15496 | CR-hvKP | BioProject PRJNA1256526 |
| HD15497 | CR-hvKP | BioProject PRJNA1256526 |
| HD15498 | CR-hvKP | BioProject PRJNA1256526 |
| HD15501 | CR-hvKP | BioProject PRJNA1256526 |
| HD15505 | CR-hvKP | BioProject PRJNA1256526 |
| HD15506 | CR-hvKP | BioProject PRJNA1256526 |
| HD15508 | CR-hvKP | BioProject PRJNA1256526 |
| HD15510 | CR-hvKP | BioProject PRJNA1256526 |
| HD15511 | CR-hvKP | BioProject PRJNA1256526 |
| HD15514 | CR-hvKP | BioProject PRJNA1256526 |
| HD15517 | CR-hvKP | BioProject PRJNA1256526 |
| HD15519 | CR-hvKP | BioProject PRJNA1256526 |
| HD15520 | CR-hvKP | BioProject PRJNA1256526 |
| HD15523 | CR-hvKP | BioProject PRJNA1256526 |
| HD15525 | CR-hvKP | BioProject PRJNA1256526 |
| HD15527 | CR-hvKP | BioProject PRJNA1256526 |
| HD15531 | CR-hvKP | BioProject PRJNA1256526 |
| HD15534 | CR-hvKP | BioProject PRJNA1256526 |
| HD15537 | CR-hvKP | BioProject PRJNA1256526 |
| HD15543 | CR-hvKP | BioProject PRJNA1256526 |
| HD15544 | CR-hvKP | BioProject PRJNA1256526 |
| HD15545 | CR-hvKP | BioProject PRJNA1256526 |
| HD15547 | CR-hvKP | BioProject PRJNA1256526 |
| HD15549 | CR-hvKP | BioProject PRJNA1256526 |
| HD15550 | CR-hvKP | BioProject PRJNA1256526 |
| HD15554 | CR-hvKP | BioProject PRJNA1256526 |
| HD15555 | CR-hvKP | BioProject PRJNA1256526 |
| HD15558 | CR-hvKP | BioProject PRJNA1256526 |
| HD15559 | CR-hvKP | BioProject PRJNA1256526 |
| HD15560 | CR-hvKP | BioProject PRJNA1256526 |
| HD15561 | CR-hvKP | BioProject PRJNA1256526 |
| HD15564 | CR-hvKP | BioProject PRJNA1256526 |
| HD15565 | CR-hvKP | BioProject PRJNA1256526 |
| HD15566 | CR-hvKP | BioProject PRJNA1256526 |
| HD15570 | CR-hvKP | BioProject PRJNA1256526 |
| HD15571 | CR-hvKP | BioProject PRJNA1256526 |
| HD15573 | CR-hvKP | BioProject PRJNA1256526 |
| HD15574 | CR-hvKP | BioProject PRJNA1256526 |
| HD15575 | CR-hvKP | BioProject PRJNA1256526 |
| HD15576 | CR-hvKP | BioProject PRJNA1256526 |
| HD15577 | CR-hvKP | BioProject PRJNA1256526 |
| HD15582 | CR-hvKP | BioProject PRJNA1256526 |
| HD12161 | CR-non-hvKP | BioProject PRJNA1256526 |
| HD12164 | CR-non-hvKP | BioProject PRJNA1256526 |
| HD12169 | CR-non-hvKP | BioProject PRJNA1256526 |
| HD12170 | CR-non-hvKP | BioProject PRJNA1256526 |
| HD12241 | CR-non-hvKP | BioProject PRJNA1256526 |
| HD12245 | CR-non-hvKP | BioProject PRJNA1256526 |
| HD12246 | CR-non-hvKP | BioProject PRJNA1256526 |
| HD12247 | CR-non-hvKP | BioProject PRJNA1256526 |
| HD12251 | CR-non-hvKP | BioProject PRJNA1256526 |
| HD12271 | CR-non-hvKP | BioProject PRJNA1256526 |
| HD12275 | CR-non-hvKP | BioProject PRJNA1256526 |
| HD12277 | CR-non-hvKP | BioProject PRJNA1256526 |
| HD12279 | CR-non-hvKP | BioProject PRJNA1256526 |
| HD12282 | CR-non-hvKP | BioProject PRJNA1256526 |
| HD12283 | CR-non-hvKP | BioProject PRJNA1256526 |
| HD12289 | CR-non-hvKP | BioProject PRJNA1256526 |
| HD12290 | CR-non-hvKP | BioProject PRJNA1256526 |
| HD12292 | CR-non-hvKP | BioProject PRJNA1256526 |
| HD12293 | CR-non-hvKP | BioProject PRJNA1256526 |
| HD12309 | CR-non-hvKP | BioProject PRJNA1256526 |
| HD12314 | CR-non-hvKP | BioProject PRJNA1256526 |
| HD12317 | CR-non-hvKP | BioProject PRJNA1256526 |
| HD12321 | CR-non-hvKP | BioProject PRJNA1256526 |
| HD12328 | CR-non-hvKP | BioProject PRJNA1256526 |
| HD12331 | CR-non-hvKP | BioProject PRJNA1256526 |
| HD12333 | CR-non-hvKP | BioProject PRJNA1256526 |
| HD12334 | CR-non-hvKP | BioProject PRJNA1256526 |
| HD12337 | CR-non-hvKP | BioProject PRJNA1256526 |
| HD12576 | CR-non-hvKP | BioProject PRJNA1256526 |
| HD13666 | CR-non-hvKP | BioProject PRJNA1256526 |
| HD13667 | CR-non-hvKP | BioProject PRJNA1256526 |
| HD13668 | CR-non-hvKP | BioProject PRJNA1256526 |
| HD13675 | CR-non-hvKP | BioProject PRJNA1256526 |
| HD13679 | CR-non-hvKP | BioProject PRJNA1256526 |
| HD13683 | CR-non-hvKP | BioProject PRJNA1256526 |
| HD13684 | CR-non-hvKP | BioProject PRJNA1256526 |
| HD13692 | CR-non-hvKP | BioProject PRJNA1256526 |
| HD13694 | CR-non-hvKP | BioProject PRJNA1256526 |
| HD13897 | CR-non-hvKP | BioProject PRJNA1256526 |
| HD13898 | CR-non-hvKP | BioProject PRJNA1256526 |
| HD13900 | CR-non-hvKP | BioProject PRJNA1256526 |
| HD13908 | CR-non-hvKP | BioProject PRJNA1256526 |
| HD13910 | CR-non-hvKP | BioProject PRJNA1256526 |
| HD13912 | CR-non-hvKP | BioProject PRJNA1256526 |
| HD13913 | CR-non-hvKP | BioProject PRJNA1256526 |
| HD13915 | CR-non-hvKP | BioProject PRJNA1256526 |
| HD13916 | CR-non-hvKP | BioProject PRJNA1256526 |
| HD13917 | CR-non-hvKP | BioProject PRJNA1256526 |
| HD13920 | CR-non-hvKP | BioProject PRJNA1256526 |
| HD13922 | CR-non-hvKP | BioProject PRJNA1256526 |
| HD13923 | CR-non-hvKP | BioProject PRJNA1256526 |
| HD13924 | CR-non-hvKP | BioProject PRJNA1256526 |
| HD13925 | CR-non-hvKP | BioProject PRJNA1256526 |
| HD13926 | CR-non-hvKP | BioProject PRJNA1256526 |
| HD13927 | CR-non-hvKP | BioProject PRJNA1256526 |
| HD13928 | CR-non-hvKP | BioProject PRJNA1256526 |
| HD13929 | CR-non-hvKP | BioProject PRJNA1256526 |
| HD13930 | CR-non-hvKP | BioProject PRJNA1256526 |
| HD13932 | CR-non-hvKP | BioProject PRJNA1256526 |
| HD13935 | CR-non-hvKP | BioProject PRJNA1256526 |
| HD13939 | CR-non-hvKP | BioProject PRJNA1256526 |
| HD13942 | CR-non-hvKP | BioProject PRJNA1256526 |
| HD13943 | CR-non-hvKP | BioProject PRJNA1256526 |
| HD13944 | CR-non-hvKP | BioProject PRJNA1256526 |
| HD13946 | CR-non-hvKP | BioProject PRJNA1256526 |
| HD13947 | CR-non-hvKP | BioProject PRJNA1256526 |
| HD13948 | CR-non-hvKP | BioProject PRJNA1256526 |
| HD13951 | CR-non-hvKP | BioProject PRJNA1256526 |
| HD13952 | CR-non-hvKP | BioProject PRJNA1256526 |
| HD13954 | CR-non-hvKP | BioProject PRJNA1256526 |
| HD13957 | CR-non-hvKP | BioProject PRJNA1256526 |
| HD13958 | CR-non-hvKP | BioProject PRJNA1256526 |
| HD13959 | CR-non-hvKP | BioProject PRJNA1256526 |
| HD13960 | CR-non-hvKP | BioProject PRJNA1256526 |
| HD13963 | CR-non-hvKP | BioProject PRJNA1256526 |
| HD14724 | CR-non-hvKP | BioProject PRJNA1256526 |
| HD14725 | CR-non-hvKP | BioProject PRJNA1256526 |
| HD14726 | CR-non-hvKP | BioProject PRJNA1256526 |
| HD14727 | CR-non-hvKP | BioProject PRJNA1256526 |
| HD14728 | CR-non-hvKP | BioProject PRJNA1256526 |
| HD14729 | CR-non-hvKP | BioProject PRJNA1256526 |
| HD14731 | CR-non-hvKP | BioProject PRJNA1256526 |
| HD14732 | CR-non-hvKP | BioProject PRJNA1256526 |
| HD14733 | CR-non-hvKP | BioProject PRJNA1256526 |
| HD14734 | CR-non-hvKP | BioProject PRJNA1256526 |
| HD14737 | CR-non-hvKP | BioProject PRJNA1256526 |
| HD14749 | CR-non-hvKP | BioProject PRJNA1256526 |
| HD14753 | CR-non-hvKP | BioProject PRJNA1256526 |
| HD14898 | CR-non-hvKP | BioProject PRJNA1256526 |
| HD14899 | CR-non-hvKP | BioProject PRJNA1256526 |
| HD14900 | CR-non-hvKP | BioProject PRJNA1256526 |
| HD14904 | CR-non-hvKP | BioProject PRJNA1256526 |
| HD14908 | CR-non-hvKP | BioProject PRJNA1256526 |
| HD14911 | CR-non-hvKP | BioProject PRJNA1256526 |
| HD14914 | CR-non-hvKP | BioProject PRJNA1256526 |
| HD14920 | CR-non-hvKP | BioProject PRJNA1256526 |
| HD14923 | CR-non-hvKP | BioProject PRJNA1256526 |
| HD14930 | CR-non-hvKP | BioProject PRJNA1256526 |
| HD14935 | CR-non-hvKP | BioProject PRJNA1256526 |
| HD14936 | CR-non-hvKP | BioProject PRJNA1256526 |
| HD14939 | CR-non-hvKP | BioProject PRJNA1256526 |
| HD14945 | CR-non-hvKP | BioProject PRJNA1256526 |
| HD15061 | CR-non-hvKP | BioProject PRJNA1256526 |
| HD15062 | CR-non-hvKP | BioProject PRJNA1256526 |
| HD15064 | CR-non-hvKP | BioProject PRJNA1256526 |
| HD15066 | CR-non-hvKP | BioProject PRJNA1256526 |
| HD15068 | CR-non-hvKP | BioProject PRJNA1256526 |
| HD15072 | CR-non-hvKP | BioProject PRJNA1256526 |
| HD15073 | CR-non-hvKP | BioProject PRJNA1256526 |
| HD15074 | CR-non-hvKP | BioProject PRJNA1256526 |
| HD15075 | CR-non-hvKP | BioProject PRJNA1256526 |
| HD15076 | CR-non-hvKP | BioProject PRJNA1256526 |
| HD15077 | CR-non-hvKP | BioProject PRJNA1256526 |
| HD15078 | CR-non-hvKP | BioProject PRJNA1256526 |
| HD15081 | CR-non-hvKP | BioProject PRJNA1256526 |
| HD15082 | CR-non-hvKP | BioProject PRJNA1256526 |
| HD15086 | CR-non-hvKP | BioProject PRJNA1256526 |
| HD15095 | CR-non-hvKP | BioProject PRJNA1256526 |
| HD15098 | CR-non-hvKP | BioProject PRJNA1256526 |
| HD15100 | CR-non-hvKP | BioProject PRJNA1256526 |
| HD15101 | CR-non-hvKP | BioProject PRJNA1256526 |
| HD15105 | CR-non-hvKP | BioProject PRJNA1256526 |
| HD15107 | CR-non-hvKP | BioProject PRJNA1256526 |
| HD15480 | CR-non-hvKP | BioProject PRJNA1256526 |
| HD15483 | CR-non-hvKP | BioProject PRJNA1256526 |
| HD15484 | CR-non-hvKP | BioProject PRJNA1256526 |
| HD15487 | CR-non-hvKP | BioProject PRJNA1256526 |
| HD15492 | CR-non-hvKP | BioProject PRJNA1256526 |
| HD15494 | CR-non-hvKP | BioProject PRJNA1256526 |
| HD15499 | CR-non-hvKP | BioProject PRJNA1256526 |
| HD15502 | CR-non-hvKP | BioProject PRJNA1256526 |
| HD15504 | CR-non-hvKP | BioProject PRJNA1256526 |
| HD15507 | CR-non-hvKP | BioProject PRJNA1256526 |
| HD15509 | CR-non-hvKP | BioProject PRJNA1256526 |
| HD15512 | CR-non-hvKP | BioProject PRJNA1256526 |
| HD15515 | CR-non-hvKP | BioProject PRJNA1256526 |
| HD15516 | CR-non-hvKP | BioProject PRJNA1256526 |
| HD15518 | CR-non-hvKP | BioProject PRJNA1256526 |
| HD15521 | CR-non-hvKP | BioProject PRJNA1256526 |
| HD15524 | CR-non-hvKP | BioProject PRJNA1256526 |
| HD15530 | CR-non-hvKP | BioProject PRJNA1256526 |
| HD15533 | CR-non-hvKP | BioProject PRJNA1256526 |
| HD15535 | CR-non-hvKP | BioProject PRJNA1256526 |
| HD15539 | CR-non-hvKP | BioProject PRJNA1256526 |
| HD15546 | CR-non-hvKP | BioProject PRJNA1256526 |
| HD15552 | CR-non-hvKP | BioProject PRJNA1256526 |
| HD15553 | CR-non-hvKP | BioProject PRJNA1256526 |
| HD15556 | CR-non-hvKP | BioProject PRJNA1256526 |
| HD15557 | CR-non-hvKP | BioProject PRJNA1256526 |
| HD15562 | CR-non-hvKP | BioProject PRJNA1256526 |
| HD15563 | CR-non-hvKP | BioProject PRJNA1256526 |
| HD15567 | CR-non-hvKP | BioProject PRJNA1256526 |
| HD15568 | CR-non-hvKP | BioProject PRJNA1256526 |
| HD15569 | CR-non-hvKP | BioProject PRJNA1256526 |
| HD15572 | CR-non-hvKP | BioProject PRJNA1256526 |
| HD15578 | CR-non-hvKP | BioProject PRJNA1256526 |
| HD15579 | CR-non-hvKP | BioProject PRJNA1256526 |
| HD15580 | CR-non-hvKP | BioProject PRJNA1256526 |
| HD15581 | CR-non-hvKP | BioProject PRJNA1256526 |
